# Synthesis of the cyanobacterial halometabolite Chlorosphaerolactylate B and demonstration of its antimicrobial effect *in vitro* and *in vivo*

**DOI:** 10.3389/fmicb.2022.950855

**Published:** 2022-09-29

**Authors:** Nikoline Jensen, Henrik Elvang Jensen, Bent Aalbaek, Sophie Amalie Blirup-Plum, Sara M. Soto, Virginio Cepas, Yuly López, Yaiza Gabasa, Ignacio Gutiérrez-del-Río, Claudio J. Villar, Felipe Lombó, María José Iglesias, Raquel Soengas, Fernando López Ortiz, Louise Kruse Jensen

**Affiliations:** ^1^Section for Pathobiological Sciences, Department of Veterinary and Animal Science, University of Copenhagen, Copenhagen, Denmark; ^2^ISGlobal, Hospital Clínic, Universitat de Barcelona, Barcelona, Spain; ^3^CIBER Enfermedades Infecciosas (CIBERINFEC), Instituto de Salud Carlos III, Madrid, Spain; ^4^Biotechnology in Nutraceuticals and Bioactive Compounds-BIONUC, Department of Functional Biology, University of Oviedo, Oviedo, Spain; ^5^Instituto Universitario de Oncología del Principado de Asturias (IUOPA), Oviedo, Spain; ^6^Instituto de Investigación Sanitaria del Principado de Asturias (ISPA), Oviedo, Spain; ^7^Área de Química Orgánica, Centro de Investigación CIAIMBITAL, Universidad de Almería, Almería, Spain

**Keywords:** halometabolites, cyanobacteria, biofilm, osteomyelitis, porcine model

## Abstract

Chlorosphaerolactylate B, a newly discovered antimicrobial halometabolite from the cyanobacterium *Sphaerospermopsis* sp. LEGE 00249 has been synthesized in three steps by using 12-bromododecanoic acid as starting material. A total of 0.5 g was produced for *in vitro* and *in vivo* antimicrobial efficacy testing. *In vitro*, the minimal inhibitory concentration (MIC) was estimated to be 256 mg/L for *Staphylococcus aureus*, while the minimal biofilm inhibitory concentration (MBIC) was estimated to be 74 mg/L. The *in vivo* study utilized a porcine model of implant-associated osteomyelitis. In total, 12 female pigs were allocated into 3 groups based on inoculum (*n* = 4 in each group). An implant cavity (IC) was drilled in the right tibia and followed by inoculation and insertion of a steel implant. All pigs were inoculated with 10 μL containing either: 11.79 mg synthetic Chlorosphaerolactylate B + 10^4^ CFU of *S. aureus* (Group A), 10^4^ CFU of *S. aureus* (Group B), or pure saline (Group C), respectively. Pigs were euthanized five days after inoculation. All Group B animals showed macroscopic and microscopic signs of bone infection and both tissue and implant harbored *S. aureus* bacteria (mean CFU on implants = 1.9 × 10^5^). In contrast, *S. aureus* could not be isolated from animals inoculated with saline. In Group A, two animals had a low number of *S. aureus* (CFU = 6.7 × 10^1^ and 3.8 × 10^1^, respectively) on the implants, otherwise all Group A animals were similar to Group C animals. In conclusion, synthetic Chlorosphaerolactylate B holds potential to be a novel antimicrobial and antibiofilm compound.

## Introduction

Chronic bacterial infections are an increasing problem across the world and infections are often described in patients with a compromised immune system and around foreign bodies like catheters, pacemakers, and orthopedic prostheses or implants (Rosman et al., 2022; Kisat and Zarzaur, 2022). Chronic infections are extremely costly and difficult to manage, and therapy involves massive and prolonged administration of antibiotics (Metsemakers et al., 2018). Therefore, treatment of chronic bacterial infections is severely affected by the widespread problems of rising antimicrobial resistance in pathogenic bacteria (Pulingam et al., 2022). Furthermore, treatment of chronic bacterial infections often fails, because of bacterial biofilm formation (Singh et al., 2002; Roberts et al., 2015; Cascioferro et al., 2021). Biofilms are complex populations of bacterial cells enclosed in extracellular polymeric matrix growing from an abiotic surface such as orthopedic implants or within tissues (Hall-Stoodley et al., 2004; Jensen et al., 2017a). Biofilms can hold many, often species specific, survival mechanisms, i.e., antibiotic efflux-pumps and impenetrable matrixes (Soto, 2013; Olsen, 2015). Bacteria growing in biofilms can be up to 1000-fold more tolerant to antibiotics than their single planktonic counterpart (Pulingam et al., 2022). Therefore, treatment of chronic biofilm-based infections contributes to an extremely high consumption of antibiotics, which is classified as a main driver of antibiotic resistance (Lau et al., 2019).

Thus, there is a great need for new treatment options to combat both antimicrobial-resistant bacteria and bacterial biofilm formation, and many approaches to discover new antimicrobial drugs are being researched. One approach is to utilize marine or freshwater organisms, such as microalgae and cyanobacteria, that produce a wide range of antibacterial and antibiofilm metabolites (Santhakumari et al., 2016; Antunes et al., 2019; López and Soto, 2019). Recently, 600 strains of microalgae and cyanobacteria were screened for antibiofilm and/or antibacterial activity carried out under the European H2020 project named NOMORFILM (Novel marine biomolecules against biofilm. Application to medical devices) (Cepas et al., 2021). In that project, a new halometabolite family of chlorinated lactylates named Chlorosphaerolactylates A–D was isolated from a methanolic extract of the cyanobacterium *Sphaerospermopsis sp.* LEGE 00249 (Gutiérrez-del-Río et al., 2020). Chlorosphaerolactylates A-D demonstrated antibacterial, antifungal and antibiofilm activity *in vitro* against *Staphylococcus aureus* (strain S54F9), *Candida parapsilosis* (SMI 416) and coagulase negative *Staphylococcus hominis* isolated from a porcine lung abscess, a human bloodstream infection and a patient with infected joint prosthesis, respectively (Gutiérrez-del-Río et al., 2020). Herein we describe the first chemical synthesis of Chlorosphaerolactylate B. Chlorosphaerolactylate B was the natural extracted halogenated lactylates with best antimicrobial (both antibiofilm and antibacterial) activity. Furthermore, we confirmed the antimicrobial activity of the synthetic compound both *in vitro* and *in vivo* by using a porcine model of implant associated osteomyelitis (IAO) infected with *S. aureus* strain S54F9 (Jensen et al., 2017b; Lüthje et al., 2020).

## Results

### Synthesis of Chlorosphaerolactylate B

**FIGURE 1:**
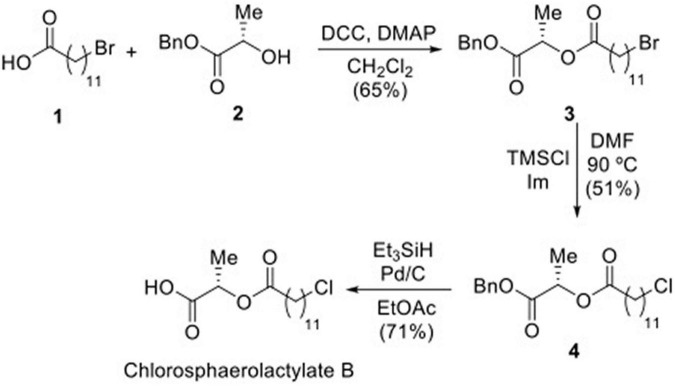
Synthesis of Chlorosphaerolactylate B naturally produced by the cyanobacterium *Sphaerospermopsis* sp. LEGE 00249 *via* intermediates **3** and **4**.

Natural Chlorosphaerolactylate B was synthesized in three steps using commercially available 12-bromododecanoic acid (Kisat and Zarzaur, 2022) as starting material (Figure 1). Coupling of **1** with L-lactic acid benzyl ester (Rosman et al., 2022), in the presence of DCC (*N,N’*-dicyclohexylcarbodiimide) as coupling agent and catalytic DMAP (dimethylaminopyridine) in DCM (dichloromethane), afforded lactylate in 65% yield (Alvarado et al., 2007; Zhu et al., 2011). Displacement of bromine in compound **3** by a chloride was achieved on reaction with TMSCl (trimethylsilyl chloride) and imidazole in DMF (dimethylformamide) at 90 °C for 7 h, affording chlorolactylate (Pulingam et al., 2022) in a yield of 51% (Peyrat et al., 1996). The substitution of the bromide atom for a chloride was deduced by the deshielding observed in the ^1^H NMR spectrum for the signal of the H-12’ from δ 3.42 (t, 2H, *J* = 6.9 Hz) ppm in compound **3** to δ 3.55 (t, 2H, *J* = 6.8 Hz) ppm in compound **4**, and further confirmed by the electrospray ionization mass spectrometry (ESI-MS) of **4**, showing a strong ion at *m/z* 3965.1985 ([M + H]^+^) and the isotopic pattern for one chloride atom. Removal of the benzyl protecting group was carried out on treatment of **4** with triethylsilane as a hydrogen source in the presence of catalytic Pd/C in ethyl acetate as solvent, to finally yield desired Chlorosphaerolactylate B in a 71% yield (Figure 1). This route allowed to prepare the desired natural product with a purity higher than 99% in a 25% overall yield. The process was scaled-up to the preparation of 0.5 g of Chlorosphaerolactylate B to be used for antimicrobial testing *in vitro* and *in vivo*. See Supplementary Figures 1–6 for ^1^H and ^13^C NMR spectra of compounds **3**, **4** and Chlorosphaerolactylate B.

### *In vitro* study of antimicrobial efficacy

Antibacterial activity: The *in vitro* MIC value of synthetic Chlorosphaerolactylate B against the *S. aureus* strain S54F9 was 256 mg/L. Positive and negative controls performed as expected, i.e., growth and no growth, respectively. Antibiofilm activity: The *in vitro* MBIC value of synthetic Chlorosphaerolactylate B against the Coagulase Negative *Staphylococcus* strain FI31 was 74 mg/L. No antibiofilm activity was registered for intermediate products (**3** and **4**). Positive and negative controls performed as expected.

### *In vivo* study of antimicrobial efficacy

**FIGURE 2:**
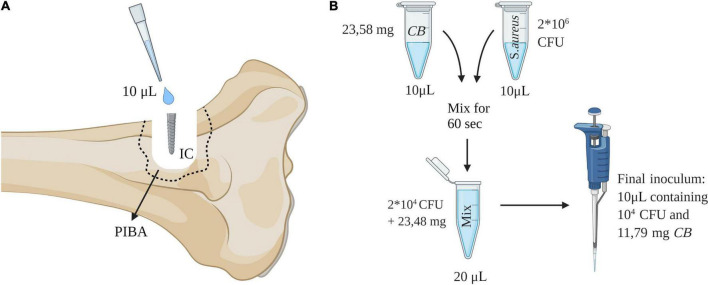
**(A)** Procedure for *in vivo* study of antimicrobial efficacy. An implant cavity (IC) was established in the right tibia of pigs by drilling. An inoculum of 10 μL was injected into the cavity and followed by insertion of a 2 × 15 mm steel implant. Pathological manifestations around IC are defined as the peri-implant pathological bone area (PIBA). **(B)** Preparation of inoculum containing synthetic Chlorosphaerolactylate B (CB). This figure was created using Biorender.com.

The experiment applied an *in vivo* susceptibility test for prevention of biofilm formation in tissue and on implants (Jensen et al., 2019). The test utilizes the porcine osteomyelitis model described by Jensen et al. (2017a,b). In this model, biofilm is understood as bacterial aggregates surrounded by extracellular matrix and the presence of biofilm has been demonstrated using electron microscopy, immunohistochemistry and *in situ* hybridization (Jensen et al., 2017a,^2018^). It has been shown several times that a fulminant bone infection with biofilm formation is formed after 5 days within the model, i.e., pathological manifestations as seen in humans with bone infections are present (Jensen et al., 2017b; Lüthje et al., 2020). The principle of the used *in vivo* susceptibility test is to mix the bacterial inoculum with active compound just before injection into a pre-drilled implant cavity (IC) of the tibial bone (Figure 2). In brief, twelve pigs were inoculated with either synthetic Chlorosphaerolactylate B + *S. aureus* (Group A), *S. aureus* (Group B), or saline (Group C), respectively (Table 1). All inoculations were successful, i.e., the protocol was fulfilled, and all animals completed the 5-day study protocol as expected. During the study period, animals did not show lameness, and all pigs eat, drank and showed normal pig behavior.

**TABLE 1:** Study design and microbiology results for *in vivo* evaluation of synthetic Chlorosphaerolactylate B in a porcine model of implant-associated osteomyelitis.

Group	Animal ID	Inoculum – 10 μL with:	Microbiology			
			
			SC	IC	Sonication (*S. aureus* -CFU/mL)	IHC detection of *S. aureus* in PIBA
A	1	*Staphylococcus aureus* 10^4^ CFU + Chlorosphaerolactylate B	*Pasteurella caballi*	Sterile	6.7 × 10^1^	No
	2		Sterile	Sterile	0	No
	3		Sterile	Sterile	0	No
	4		Sterile	Sterile	8.33 × 10^1^	No
B	1	*S. aureus* 10^4^ CFU	*S. aureus*	*S. aureus*	2.68 × 10^5^	Yes
	2		*S. aureus*	*S. aureus*	2.68 × 10^5^	Yes
	3		*S. aureus*	*S. aureus*	1.2 × 10^5^	Yes
	4		*S. aureus*	*S. aureus*	1.2 × 10^5^	Yes
C	1	Sterile saline	*Escherichia. coli*	*E. coli*	0	No
	2		*Aerococcus viridans*	Sterile	0	No
	3		*P. caballi*	*Streptococcus mitis*	0	No
	4		Sterile	Sterile	0	No

Microbiology studies were based on swabs from subcutis (SC) and implant cavity (IC), sonication of implants, and immunohistochemistry (IHC) toward *S. aureus* of the peri-implant pathological bone area (PIBA). CFU, Colony forming unit.

#### Gross pathology

**FIGURE 3:**
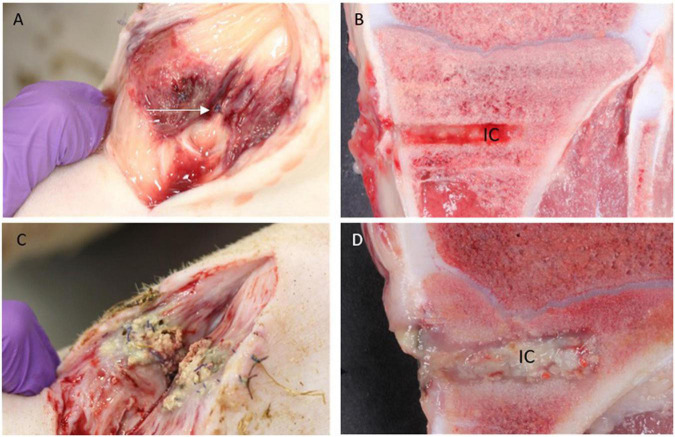
Macroscopic pathology. **(A,B)**: Pig inoculated with *S. aureus* + Chlorosphaerolactylate B in the tibial bone. Pus is not seen subcutaneously, and the periosteal sutures are intact (arrow). The red color represents inflammation related to the surgical procedure *per se*. In the bone, the implant cavity (IC) is regular and without pus. **(C,D)**: Pig inoculated with *S. aureus* only. Pus is present both in subcutis and within IC.

Two Group A animals showed signs of infection, i.e., minor accumulations of pus in the operation wound adjacent to IC, while this occurred in all Group B animals (Figure 3). Only one animal from Group C showed pus in relation to the surgical wound. Pus was not identified around the implant in any Groups A and C animals, while all 4 animals of Group B had pus present around the implant (Figure 3). Multiplication of length and width of the regional inguinal lymph nodes demonstrated local lymph node enlargement localized to the implant side in comparison to control side in all animals. However, the biggest enlargement was present within Group B. No other organs demonstrated gross lesions.

#### Microbiology

Results of local microbiology are reported in Table 1. *Staphylococcus aureus* was reisolated from all Group B pigs despite test type. The sonication results showed a high bacterial load on the implant surface in Group B animals. The bacterial implant load was either reduced by a log-factor 4 or totally absent when synthetic Chlorosphaerolactylate B was administrated (Table 1). Bacterial growth on implants could not be demonstrated in any Group C animals. Systemic spreading of bacteria was not observed, i.e., all caudal lung lobes were found sterile. Contamination of the surgical site with other bacteria than *S. aureus* was observed in Groups A and C (Table 1). The contamination originates from the skin of pigs and the stable environment.

#### Histology and immunohistochemistry

**FIGURE 4:**
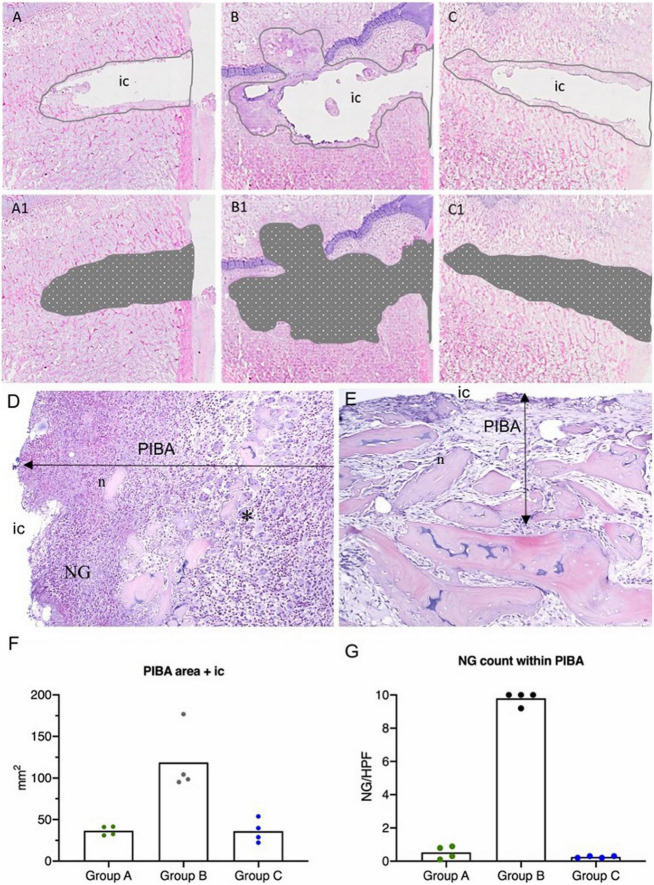
Results of histology in a porcine model of implant-associated osteomyelitis (IAO) evaluating the *in vivo* antimicrobial effect of Chlorosphaerolactylate B. **(A–C)**: Estimation of peri-implant pathological bone area (PIBA). The lines are drawn at the junction of normal bone pattern and pathological manifestations. Picture **(A)** is from a pig inoculated with *S. aureus* and Chlorosphaerolactylate B, picture **(B)** is from a pig inoculated with *S. aureus*, and picture **(C)** is from a pig inoculated with saline. **(A1,B1,C1)**: The area of PIBA including the implant cavity (ic). **(D)**: Pig inoculated with *S. aureus* only. PIBA shows high cellularity, mostly consisting of macrophages and neutrophil granulocytes (NG). Necrotic bone (n) and cellular debris are located adjacent to IC. Many giant cells consistent with osteoclasts (*) can be found in PIBA. HE X 100. **(E)**: Pig inoculated with *S. aureus* and Chlorosphaerolactylate B. Necrotic bone trabecula (n) and cellular debris were seen adjacent to IC. PIBA had relatively low cellularity. HE X 100. **(F)**: Area of PIBA in each animal (mm^2^). **(G)**: Average number of Neutrophils (NG)/High Power Field (HPF) within PIBA in each animal. Boxes represent group means.

**FIGURE 5:**
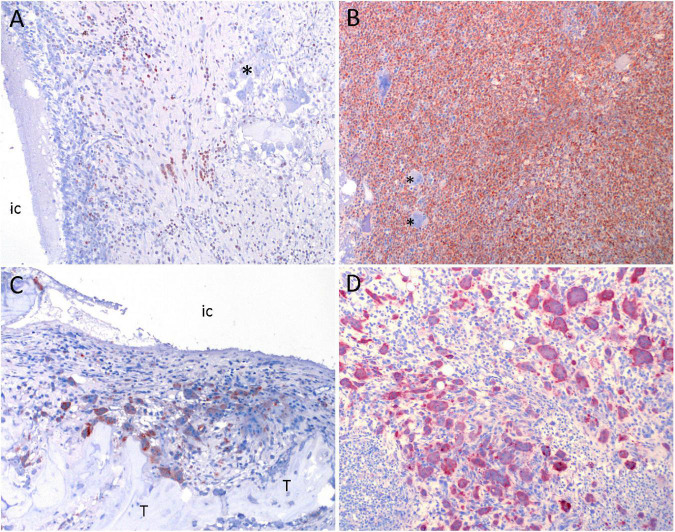
Results of immunohistochemistry in a porcine model of implant-associated osteomyelitis (IAO) evaluating the *in vivo* antimicrobial effect of Chlorosphaerolactylate B. **(A,B)**: MAC-387 positive cells (macrophages and neutrophils) are red. Picture **(A)** is from a pig inoculated with Chlorosphaerolactylate B + *S. aureus*. Picture **(B)** is from a pig inoculated with *S. aureus* and shows massive amounts of MAC-387 positive cells. **(C, D)**: Cathepsin K positive cells (osteoclastic precursor cells or mature osteoclasts) are red. Picture **(C,D)** is from the same pigs as shown in **(A,B)**. Picture **(D)** shows massive amounts of Cathepsin K positive cells. *, unstained multinucleated giant cells; IC, implant cavity; T, live trabecula.

The estimated size of the peri-implant pathological bone areas (PIBA) in Groups A and C was low with group means of 36.66 mm^2^ and 36.21 mm^2^, respectively (Figure 4). In these groups, PIBA was easily defined, and consisted of manifestations related to drilling and a normal healing response, i.e., necrotic bone debris, varying degrees of fibroplasia, edema and macrophages (Figure 4). Only few neutrophils (NG) were present and the cellularity of the lesions was generally low. In contrast, Group B animals were characterized by having large PIBA values (group mean of 118.73 mm^2^). In Group B, PIBA consisted of bone necrosis and accumulations of inflammatory cells, and an irregular outline of the IC was observed (Figure 4). The inflammatory cell population was dominated by macrophages and neutrophils, and sometimes microabscesses were found (massive accumulations of neutrophil granulocytes). In all groups, the identity of macrophages and neutrophils were confirmed with positive MAC-387 IHC staining (Figure 5). Estimations of the NG counts within PIBA showed that slides from Groups A and C contained very few NGs with group means of 0.53 and 0.25 NG/high power field (HPF), respectively (Figure 5). Group B had 10 NGs/HPF in three out of four cases, and the group mean was 9.8 NGs/HPF (Figure 5). Giant-cells (multinucleated cells) were present in high numbers around bone trabecula and within foci of inflammatory cells of Group B (Figure 5), and only sporadically identified within PIBA of Groups A and C, respectively. Multinucleated giant cells were positive for Cathepsin K and without positive MAC-387 IHC staining (Figure 5). This staining pattern is consistent with osteoclastic precursor cells or mature osteoclasts. Coccoid bacterial colonies positive for *S. aureus* with IHC could only be identified within Group B (Table 1). The colonies were generally localized to the middle part of PIBA. All positive and nonsense IHC controls performed as expected, i.e., with positive staining and no staining, respectively. All left control tibias showed no lesions and no signs of cell damage or inflammation were seen in the liver, kidney, lung, or spleen in any of the animals within the three groups.

## Discussion

Chlorosphaerolactylate B is a newly discovered ester of monochlorinated lauric acid and lactic acid extracted from the freshwater cyanobacterium *Sphaerospermopsis sp*. LEGE 00249 (Gutiérrez-del-Río et al., 2020). The present study describes the first protocol for chemical synthesis of Chlorosphaerolactylate B together with examination of its antimicrobial efficacy *in vitro* and *in vivo*. In general, halogenated compounds and fatty acids are recognized for their respective antimicrobial potentials (Kasanah and Triyanto, 2019; Kumar et al., 2020). The *in vitro* antimicrobial efficacy of synthetic Chlorosphaerolactylate B was found to be related to the presence of both molecular components, i.e., the chloride atom and the hydroxycarbonyl group in the molecule. Analogously to the natural product, synthetic Chlorosphaerolactylate B was active against *S. aureus* with an observed MIC value of 256 mg/L. As far as bacterial biofilm formation was studied, the synthetic Chlorosphaerolactylate B showed a MBIC value of 74 mg/L against *S. aureus*. Thus, the MIC and MBIC values of the synthetic compound were found lower than the values observed for the natural product (1024 and 306.8 mg/L, respectively) (Gutiérrez-del-Río et al., 2020). This difference may be attributed to the mixture of potential isoforms in the natural version of Chlorosphaerolactylate B (Gutiérrez-del-Río et al., 2020). The present intermediate products **3** and **4** showed no antibiofilm activity. This indicates that the presence of the chlorine atom together with a free carboxylic acid moiety are mandatory for the antibiofilm activity of Chlorosphaerolactylate B. Other products tested against the used *S. aureus* strain entails only the aminoglycoside gentamicin which is commonly used in revision surgery of bone infections in humans (McNally et al., 2016). In a study based on the porcine IAO model gentamicin was used as active ingredient in a local bone void filler following revision surgery (Blirup-Plum et al., 2020). However, a week after revision the infection was reestablished properly due to remnant biofilms. This underline the need for development of more biofilm efficient antibiotics.

*In vivo*, synthetic Chlorosphaerolactylate B completely prevented osteomyelitis in two animals (both bone tissue and implants were found sterile), while the remaining two Group A pigs showed no bacterial bone growth and a 4 log-reduction of the implant bacterial load in comparison to Group B. Most preclinical *in vivo* studies aiming to investigate the prophylactic effect of antimicrobial interventions show a 0.3–4.5 log-reduction in tissue and implant CFU number (Masters et al., 2019). All pigs inoculated with *S. aureus* only (Group B) developed osteomyelitis and showed manifestations confirmatory with the disease in humans, e.g., pus surrounding the implant and more than five NG/HPF on average at histology (Metsemakers et al., 2018; Hofstee et al., 2020). The 100% infection rate of the porcine model make the observed *in vivo* antimicrobial efficacy of synthetic Chlorosphaerolactylate B highly reliable. The synthetic Chlorosphaerolactylate B concentration used *in vivo* was much higher than the estimated MBIC value. The used concentration of 159.346,8 times the MBIC value was chosen based on the original data from the applied *in vivo* biofilm susceptibility study (Jensen et al., 2019). In that study, 160.000 times the MIC value for gentamicin was needed to prevent bacterial growth on both the implant surface and within the surrounding tissue of the porcine IAO model (Jensen et al., 2019). Therefore, the present study aimed to use the highest possible concentration of active compounds to be mixed into the inoculum as a starting point for evaluation of antimicrobial efficacy. Pre-inoculation mixing of the inoculum with synthetic Chlorosphaerolactylate B secured an optimal and reproducible contact between bacteria and drug within the tissue. Thus, the observed antimicrobial effect of synthetic Chlorosphaerolactylate B was based on exposure to a single dose and under the influence of *in vivo* specific factors, like bleeding and inflammation development.

Natural halometabolites are formed during geothermal processes such as volcano, hot springs, or earthquakes (abiogenic) or produced by bacteria, fungi, plants, marine invertebrates, macroalgae, microalgae and cyanobacteria (biogenic) (Lee et al., 2010; Singh et al., 2017; Gribble, 2018; Kasanah and Triyanto, 2019). Halometabolites are defined as a group of compounds that contain halogen substituents (F, Cl, Br, I) and currently more than 5000 halogenated compounds have been isolated (Atashgahi et al., 2018). Biogenic halometabolites have several functions in physiological, biochemical, or defensive role for their host including communication (quorum sensing) and production of growth hormones, sex pheromones, toxins, or antibiotics (Kasanah and Triyanto, 2019). Chlorinated antibiotics were first discovered from the examination of soil actinobacteria with the discovery of streptomycin from *Streptomyces griseus* (Kasanah and Triyanto, 2019). Since then, several chlorinated antibiotics, have been discovered and many of them are available on the market, i.e., chloramphenicol and vancomycin (Kasanah and Triyanto, 2019). Vancomycin is a halogenated glycopeptide with activity against many human pathogens including *Staphylococcus aureus* and *S. epidermidis* (Aggarwal et al., 2022). Herein we confirmed that Cyanobacteria (photosynthetic prokaryotes) represent a source for identification of new types of halogenated compounds with antimicrobial properties, i.e., halogenated fatty acids. A recent study demonstrated that microalgae and cyanobacteria produce a great variety of different lipids with antibiotic and antibiofilm activity against the most important pathogens causing severe infections in humans (Cepas et al., 2021). In conclusion, the cyanobacterial halometabolite Chlorosphaerolactylate B can be synthesized, and the synthetic compound shows antibacterial and antibiofilm activity. The present study is descriptive and hypothesis generating. It represents a reliable foundation for further investigations of this new discovered type of antimicrobials. However, more *in vivo* studies of synthetic Chlorosphaerolactylate B are required in order to disclose dose-response profile, long-time exposure effects or understanding the mechanisms of actions. Chlorosphaerolactylate B hold potential to be used as an antimicrobial agent either as an alternative to current compounds for treatments or as adjuvant that could enhance biofilm eradication efficacy.

## Materials and methods

### Equipment and solvents for synthesis of Chlorosphaerolactylate B

Solvents and reagents were used as received. TLC was performed on Merck plates with aluminum backing and silica gel 60 F254. For column chromatography silica gel 60 (40–63 μm) from Scharlau was used. NMR spectra were measured on a Bruker Avance III HD 300 (^1^H, 300.13 MHz; ^13^C, 75.47 MHz) using the chemical shift of the residual solvent signal with respect to tetramethylsilane as internal reference for both nuclei. Chemical shifts are reported in parts per million (ppm, δ) and coupling constants are reported as Hertz (Hz). High resolution mass spectra were recorded on Agilent Technologies LC/MSD TOF instrument using electrospray ionization.

Benzyl (*S*)-2-[(12-bromododecanoyl)oxy]propanoate: To a solution of 12-bromododecanoic acid **1** (0.50 g, 1.79 mmol) in dry dichloromethane (6 mL), benzyl L-lactylate **2** (0.47 g, 2.67 mmol), DMAP (23 mg, 0.19 mmol) and DCC (0.41 g, 1.98 mmol) were sequentially added and the reaction mixture was stirred for 6 h at room temperature. The precipitated dicyclohexyl urea was removed by filtration over a celite pad and eluting with ethyl acetate (50 mL). The filtrate was washed with aq. 0.5 M HCl (2 × 50 mL) and aq. saturated HNaCO_3_ (2 × 50 mL), dried (Na_2_SO_4_), filtered and concentrated under reduced pressure. The resulting residue was purified by flash column chromatography (ethyl acetate/hexane 1:40) to afford benzyl bromopropanoate **3** as a yellow oil (0.51 g, 65%). ^1^H NMR (300 MHz, CDCl_3_) δ 1.29-1.46 (m, 14H), 1.51 (d, 3H, *J* = 7.1 Hz), 1.65 (p, 2H, *J* = 7.3 Hz), 1.87 (p, 2H, *J* = 6.9 Hz), 2.36-2.42 (m, 2H), 3.42 (t, 2H, *J* = 6.9 Hz), 5.12-5.24 (m, 3H), 7.34-7.38 (m, 5H) ppm. ^13^C NMR (75.5 MHz, CDCl_3_) δ 16.9, 24.8, 28.8, 29.0, 29.2, 29.37, 29.41, 29.5, 32.8, 34.0, 34.1, 45.2, 66.9, 68.4, 128.1, 128.4, 128.6, 135.4, 170.8, 173.2 ppm. MS (QTof +): 443 (97%), 442 (16%), 441 (100%). HRMS calculated for C_22_H_34_^79^BrO_4_ [M + H]^+^: *m/z* 441.1635; found: *m/z* 441.1638.

Benzyl (*S*)-2-[(12-chlorododecanoyl)oxy]propanoate (Pulingam et al., 2022): Benzyl propanoate **3** (0.51 g, 1.17 mmol) was treated with imidazole (0.16 g, 2.34 mmol), TMSCl (0.19 mL, 1.51 mmol) and DMF (0.20 mL) and heated at 90 °C in a sealed tube for 7 h. The reaction mixture was filtered over a pad of silica gel eluting with dichloromethane. After evaporation of the solvents, the mixture was purified by flash column chromatography (ethyl acetate/hexane 1:40) to afford benzyl chloropropanoate **4** as a yellow oil (0.23 g, 51%). ^1^H NMR (300 MHz, CDCl_3_) δ 1.29–1.46 (m, 14H), 1.51 (d, 3H, *J* = 7.1 Hz), 1.62–1.67 (m, 2H), 1.74–1.83 (m, 2H), 2.36–2.40 (m, 2H), 3.55 (t, 2H, *J* = 6.8 Hz), 5.12–5.24 (m, 3H), 7.34–7.39 (m, 5H) ppm. ^13^C NMR (75.5 MHz, CDCl_3_) δ 16.9, 24.8, 26.9, 28.9, 29.0, 29.2, 29.37, 29.43, 29.45, 32.6, 33.9, 45.2, 66.9, 68.4, 128.1, 128.4, 128.6, 135.4, 170.8, 173.2 ppm. MS (QTof +): 397 (100%), 398 (24%), 399 (32%), 400 (8%). HRMS calculated for C_22_H_34_^35^ClO_4_ [M + H]^+^: *m/z* 397.2127; found: *m/z* 397.2140.

(*S*)-2-[(12-Chlorododecanoyl)oxy]propanoic acid (Chlorosphaerolactylate B): To a suspension of benzyl propanoate **4** (0.23 g, 0.60 mol) and Pd/C (0.04 g, 10% wt) in degassed ethyl acetate (15 mL), triethylsilane was added (0.48 mL, 3.00 mmol) and the mixture was stirred at room temperature overnight. After filtration over celite and evaporation of the solvents in the rotary evaporator, the residue was purified by flash column chromatography (ethyl acetate/hexane 1:1) to yield Chlorosphaerolactylate B as a white solid (0.13 g, 71%). ^1^H NMR (CDCl_3_, 300 MHz) δ 1.27-1.44 (m, 14H), 1.53 (d, 3H, *J* = 7.1 Hz), 1.64 (p, 2H, *J* = 7.5 Hz), 1.72-1.81 (m, 2H), 2.35-2.40 (m, 2H), 3.53 (t, 2H, *J* = 6.8 Hz), 5.11 (q, 1H, *J* = 7.1 Hz) ppm. ^13^C NMR (CDCl_3_, 75.5 MHz) δ 16.9, 24.8, 26.9, 28.9, 29.0, 29.2, 29.38, 29.43, 29.45, 32.7, 33.9, 45.2, 67.9, 173.3 ppm. The NMR data are in excellent agreement with those reported in the literature for the natural product (Gutiérrez-del-Río et al., 2020).

### *In vitro* study of antibacterial effect

Minimal inhibitory concentration (MIC) values for synthetic Chlorosphaerolactylate B against *S. aureus* S54F9, spa type t1333 (strain used *in vivo*) were estimated, using the broth microdilution method based on the guidelines from the Clinical and Laboratory Standards Institute (CLSI), with minor modifications (CLSI, 2020). Serial two-fold dilutions of the molecules ranging from 512 to 1 mg/L were performed in a U-bottomed 96-well microtiter plate (Thermo Fisher scientific, Barcelona, Spain), and the MIC protocol was performed as in Cepas et al. (2021). Positive and negative controls were included. MIC values were defined as the lowest concentration that inhibited visible growth. The experiment was carried out by triplicate.

The antibiofilm activity of synthetic *Chlorosphaerolactylate B* and the 2 intermediate products **3** and **4** was estimated using the biofilm-forming Coagulase Negative *Staphylococcus* strain FI31. Serial two-fold dilutions of each molecule ranging from 1024 to 0.5 mg/L were performed in flat-bottomed 96-well microtiter plates (Thermo Fisher scientific, Barcelona, Spain). TBS with 0.25% of glucose was added to enhance biofilm formation. A bacterial suspension was prepared from overnight culture and adjusted to a 0.5 McFarland and consequently diluted to reach an inoculum of 50 μL bacterial suspension (5 × 10^6^ CFU) per well. Positive and negative controls were included. After 48 h incubation at 37 °C, all wells were rinsed three times with PBS to discard planktonic cells and dried at 65 °C. Biofilm formation were fully covered with a 1% Crystal Violet (CV) stain solution for 10 min at room temperature (Stepanović et al., 2007). CV was removed, and biofilms washed with PBS to eliminate excess of dye and heat-dried for 60 min. Biofilm formation was quantified by eluting the CV fixed to the biofilm in 33% glacial acetic acid and measuring absorbance of each well at 580 nm using a microplate spectrophotometer. MBIC was defined as the minimal concentration of the compound that led to a three-fold decrease in absorbance when compared to the growth control values. The experiment was carried out by triplicate.

### *In vivo* study of antibacterial effect

The experiment utilized the porcine IAO model described by Jensen et al. (2017a,b). Therefore, anesthesia, analgesia, and surgical procedures were done as previously described (Jensen et al., 2017b). Twelve female, Specific Pathogen Free, Danish Landrace pigs with a bodyweight of 30–40 kilograms (age approximately 3 months) were used. Only female pigs were used in order to reduce variance within and between groups. Pigs were allocated into 3 groups based on inoculum (Table 1). Group B and C were historic controls in agreement with the 3R guidelines (Russell et al., 1959), i.e., minimize (reduce) the number of experimental animals as much as possible. In anesthetized pigs, an implant cavity (IC) of 4 × 20 mm was established 1 cm distal to the proximal growth plate of the right tibia using an electrical drill. 10 μL of inoculum was placed in IC followed by insertion of a 2 × 15 mm steel implant. Afterward the periosteum, soft tissue and skin were closed. All pigs were euthanized 5 days post inoculation with an overdose of intravenous pentobarbital 20%. The experiment was approved by the Danish Animal Experiments Inspectorate (license No. 2013/15-2934-00946).

### Preparation of inoculum

The porcine *S. aureus* strain S54F9 [SPA-type t1333 and multi-locus sequence type (MLST) ST433] was used for inoculation in Groups A and B (Aalbæk et al., 2015). The *S. aureus* originated from a porcine lung abscess and has previously been used to model human osteomyelitis in pigs (Aalbæk et al., 2015). The strain is highly virulent and has genes encoding several toxins, including phage-associated enterotoxin, exotoxins and superantigen (Aalbæk et al., 2015). Furthermore, the strain produces biofilm (Jensen et al., 2018). Group B received a bacterial inoculum of 10^4^ CFU in 10 μL as described by Johansen et al. (2011). Likewise, a *S. aureus* suspension of 10 μL containing 2 × 10^6^ CFU was prepared for Group A. The amount of synthetic Chlorosphaerolactylate B to be used *in vivo* was 283 mg in a liquid form (120 μL), i.e., 2.36 mg/μL. From this solution 10 μL was added to the bacterial solution for Group A and mixed for 60 s. Afterward, 10 μL of this mixing solution (containing 11.79 mg Chlorosphaerolactylate B and 10^4^ CFU) was used for inoculation in Group A animals (Jensen et al., 2019). A schematic overview of the mixing procedure is shown in Figure 2.

### Gross pathology

Following euthanasia, all thoracal and abdominal organs were inspected for gross pathology. The surgical wounds and the soft tissues covering the IC were inspected for signs of inflammation and infection. The right tibia was taken out and the implant removed followed by sagittal sectioning through IC to allow for macroscopic examination and sampling for histology. The size (length and width) of the largest local lymph node, i.e., the left and right *lnn. inguinales profundi* was measured. Tissue samples from the left tibia (corresponding to the place for inoculation on the right tibia), liver, right kidney, lungs, and spleen were sampled for histopathological evaluation of infection, inflammation, and cellular damage (see histology).

### Microbiology

During necropsies, a swab was taken from IC and adjacent subcutis, respectively. Swabs were inoculated on blood agar supplied with 5% sterile bovine blood and incubated at 37°C for 24 h. Isolates were identified by matrix-assisted laser desorption ionization-time of flight (MALDI-TOF) mass spectrometry (Vitek MS RUO, bioMérieux, France). All implants were placed in cryotubes with 1.5 ml 0.9% sterile saline and kept on ice until sonication. The implants were placed in an ultrasound bath, sonicated for 5 min, diluted, plated out on blue agar plates, and incubated for two days before determination of CFU/mL (van Gennip et al., 2012). The detection limit for sonication was 50 CFU/mL. A piece of the right caudal lung lobe was also sampled for microbiology for evaluation of systemic infection (Soerensen et al., 2012).

### Histology

Tibial bones were formalin fixed (one week) and decalcified in 22% formic acid for 4 weeks. A slide of 3–4 mm covering IC (and corresponding area on the left tibia) was dehydrated in graded concentrations of ethanol, embedded in paraffin and cut into sections of 4–5 μm (Jensen et al., 2017b). Soft tissue samples from liver, kidney and lungs were also fixed in formalin for 7 days followed by dehydration, paraffin embedding and cutting. All right tibial samples were stained with hematoxylin and eosin (HE) and Masson Trichome. All other tissues were only HE stained. Slides from the right tibia was evaluated by localizing IC and Peri-Implant pathological Bone Area (PIBA). PIBA was defined as the distance from IC to the normal pattern of trabecular bone and bone marrow (Jensen et al., 2017b). The PIBA area was outlined and measured (mm^2^) using the Freehand Drawing software in DeltaPix InSight (Smørum, Denmark). In addition, the patho-morphology of PIBA was evaluated, i.e., presence of necrosis, bacteria, neutrophil granulocytes (NG), macrophages, presence of microabscesses, and fibroplasia. A specific count of NGs was obtained according to Morgenstern et al. (2018). Ten non-overlapping high-power fields (HPFs) (×400 magnification) with high cellular density were identified in each slide. In cases with few or no areas with high cellular density, 10 non-overlapping HPFs were randomly chosen. A maximum of 10 unmistakable NGs, i.e., clear neutrophil morphology, were counted in each HPF. An average NG count/HPF was calculated for each animal. Lung, liver, kidney, and spleen slides were evaluated for infection and inflammation in terms of signs of necrosis and cellular degeneration, systemic spreading of the inoculated bacteria and infiltration of inflammatory cells.

### Immunohistochemistry

Immunohistochemistry (IHC) based on primary antibodies toward *S. aureus*, MAC387, and cathepsin K was used for *in situ* identification of *S. aureus*, monocytes/macrophages/granulocytes and osteoclasts, respectively (Table 2). The paraffin-embedded tissue blocks of right tibias were deparaffinized and cut into sections of 2-3μm. TED-buffer pH 9 was used for antigen retrieval for MAC387 and cathepsin K, citrate buffer was used for *S. aureus.* After washing in TBS solution (2 × 5 min), the tissue samples were subjected to blocking of endogenous peroxidases in 0.6% H_2_O_2_ for 15-20 min. Afterward, the samples were subjected to UltraVision Protein Block (AH Diagnostics) for 5 min to prevent additional unspecific staining. Immunostaining was carried out using the UltraVision indirect Horse Radish Peroxidase (HRP) polymer-amplification technique. After 30 min of incubation with HRP-polymer (LabVision) and subsequent washing in TBS, the slides were incubated with a red chromogen solution (AEC from LabVision) and washed in distilled water. All stains were counterstained with Mayers hematoxylin, washed with water, and finally mounted in glycerol-gelatin. Positive and nonsense controls were included (Table 2). *S. aureus* stained slides were evaluated for presence of positively stained bacterial colonies, MAC387 and Cathepsin stained slides were evaluated for the amount (no, little, moderate, high) and distribution of positive cells.

**TABLE 2:** Immunohistochemistry summary.

Detection of:	Antibody type	Clone no	Manufacture, catalog no	Positive control	Non-sense control
*S. aureus*	Monoclonal, mouse	704	Abcam, ab37644, Cambridge, United Kingdom	Porcine lung with known *S. aureus* infection	Monoclonal, mouse, IgG3, Bio-rad, MCA 2063, Kingston, United Kingdom
Macrophages/ Neutophils	Monoclonal, mouse	Mac-387	Bio-rad, MCA874G, Kingston, United Kingdom	Porcine lymph node	Monoclonal Mouse, IgG1 Agilent, X0931, Glostrup, DK
Osteoclasts	Polyclonal, rabbit	–	BioVision^3^, 3588, Mipitas, CA	Porcine bone sample	Polyclonal, rabbit, Agilent, X0903, Glostrup, DK

Antibodies used for *in vivo* testing of Chlorosphaerolactylate B in a porcine model of implant-associated osteomyelitis.

### Statistics

Obtained data from the different groups of the *in vivo* study were evaluated by calculation of mean values and standard deviations together with graphical presentation of all data points. All calculations and graphic presentation of obtained data were performed using GraphPad Prism version 9.2.0 (La Jolla, CA, United States).

## Data availability statement

The original contributions presented in this study are included in the article/Supplementary material, further inquiries can be directed to the corresponding authors.

## Ethics statement

This animal study was reviewed and approved by Danish Animal Experiments Inspectorate (license No. 2013/15-2934-00946).

## Author contributions

HJ, YL, SS, VC, YG, IG-d-R, CV, FL, MJI, RS FLO, and LJ were all partners of the EU Horizon 2020 project called NOMORFILM and the present study was designed as a part of the NOMORFILM project. MJI, RS, and FLO synthesized Chlorosphaerolactylate B. SS, VC, YL, and YG performed MBIC analysis. IG-d-R, CV, and FL performed MIC analysis. NJ, LJ, HJ, BA, and SB-P performed the *in vivo* porcine study including postmortem pathology and microbiology. NJ, SS, FL, FLO, and LJ drafted the manuscript. All authors read and commented on the manuscript.
