# Supplementary material for: Synthesis of the cyanobacterial halometabolite Chlorosphaerolactylate B and demonstration of its antimicrobial effect in vitro and in vivo
Source: Front Microbiol. 2022 Sep 29;13:950855. doi: 10.3389/fmicb.2022.950855 (PMC9557163; doi:10.3389/fmicb.2022.950855)
Supplement: Supplementary file 1 [file Table_1.DOCX]

Supplementary Material

# Supplementary Figures

**Contents**

**Figure S1**. ^1^H NMR spectrum (300 MHz, CDCl_3_) of compound **3** S2

**Figure S2**. ^13^C NMR spectrum (75.5 MHz, CDCl_3_) of compound **3** S2

**Figure S3**. ^1^H NMR spectrum (300 MHz, CDCl_3_) of compound **4** S3

**Figure S4**. ^13^C NMR spectrum (75.5 MHz, CDCl_3_) of compound **4** S3

**Figure S5**. ^1^H NMR spectrum (300 MHz, CDCl_3_) of chlorosphaerolactylate B S4

**Figure S6**. ^13^C NMR spectrum (75.5 MHz, CDCl_3_) of chlorosphaerolactylate B S4


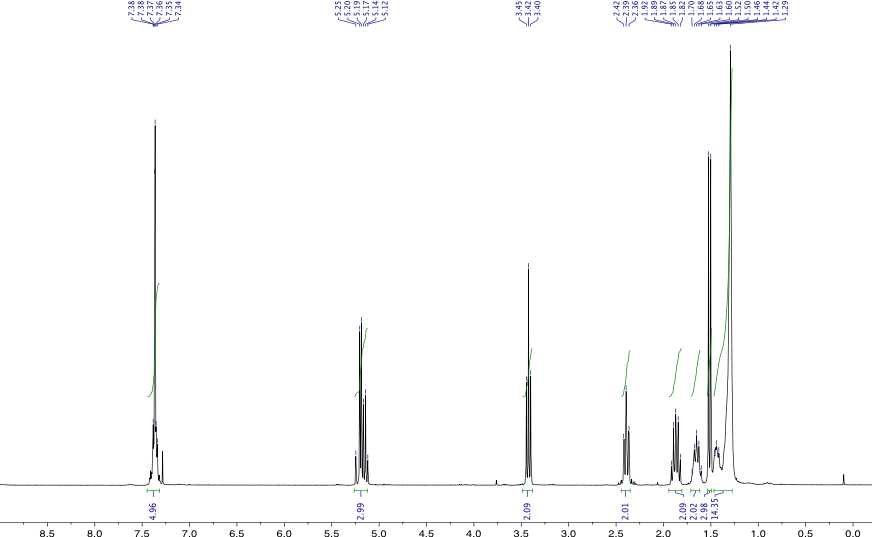


**Figure S1**. ^1^H NMR spectrum (300 MHz, CDCl_3_) of compound **3**.


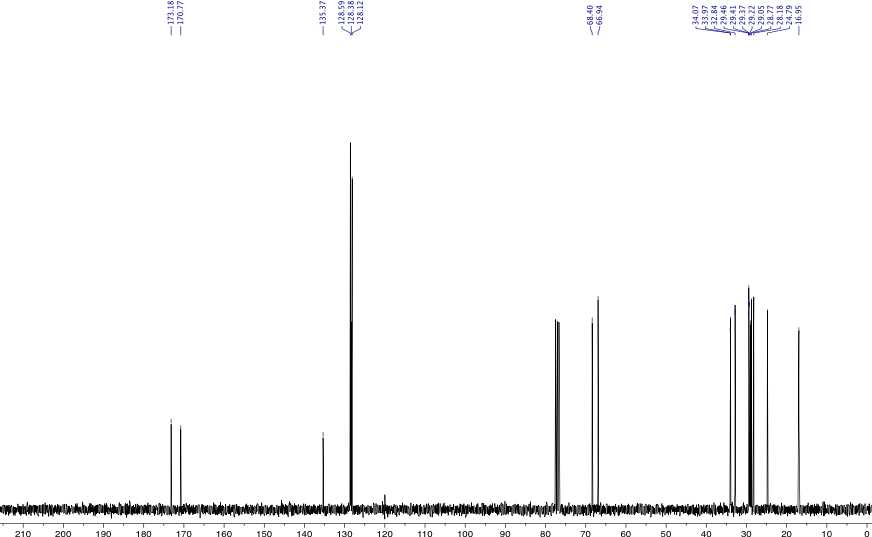


**Figure S2**. ^13^C NMR spectrum (75.5 MHz, CDCl_3_) of compound **3**.


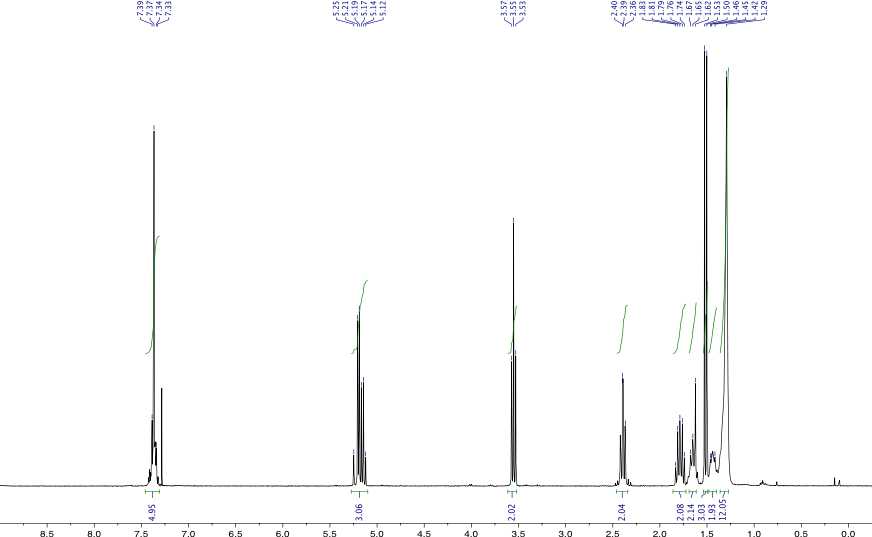


**Figure S3**. ^1^H NMR spectrum (300 MHz, CDCl_3_) of compound **4**.


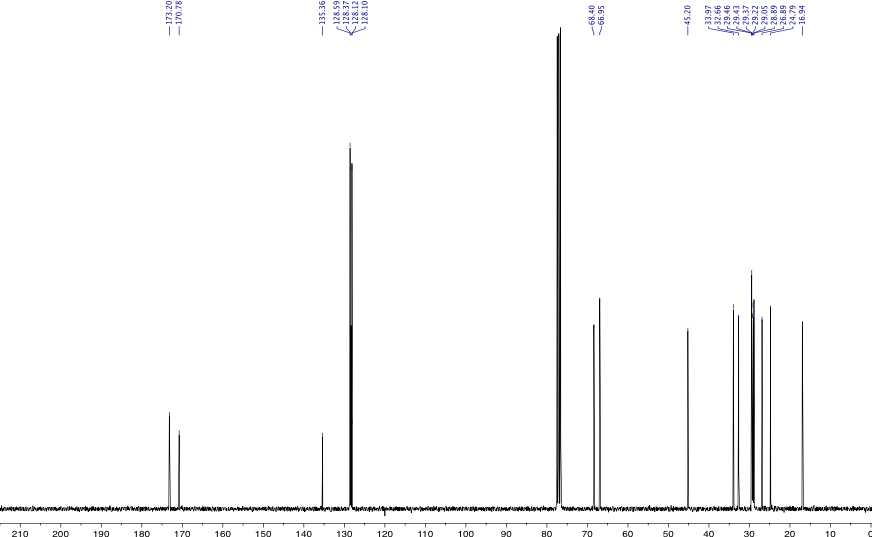


**Figure S4**. ^13^C NMR spectrum (75.5 MHz, CDCl_3_) of compound **4**.


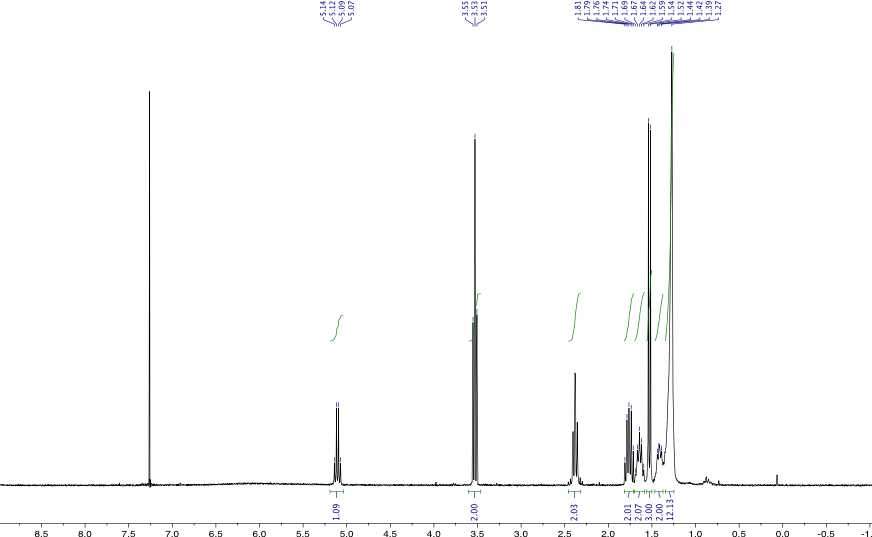


**Figure S5**. ^1^H NMR spectrum (300 MHz, CDCl_3_) of chlorosphaerolactylate B.


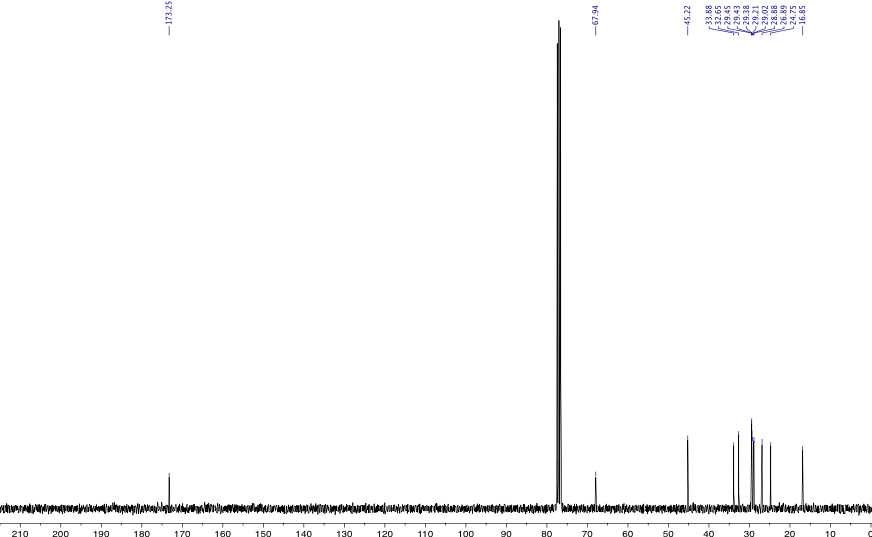


**Figure S6**. ^13^C NMR spectrum (75.5 MHz, CDCl_3_) of chlorosphaerolactylate B.
