# Supplementary material for: Synthesis of the cyanobacterial halometabolite Chlorosphaerolactylate B and demonstration of its antimicrobial effect in vitro and in vivo
Source: Front Microbiol. 2022 Sep 29;13:950855. doi: 10.3389/fmicb.2022.950855 (PMC9557163; doi:10.3389/fmicb.2022.950855)
Supplement: Supplementary file 2 [file Table_2.DOCX]

**Revision letter**

Dear reviewers and editor

Thank you for the positive criticism of our paper. We have answered all comments and suggestions and we really think that they have contributed to improve the manuscript.

.

Reviewer 1:

One of the limitations is that the antibiofilm and antimicrobial effect of the compounds are often not well separated. The authors should present more results demonstrating the in vitro results (a figure for instance) for both the antimicrobial and antibiofilm results including the values for the controls.

Very good and relevant comment. The difference between antibiofilm and antibacterial has been emphasized in the end of the introduction and under *in vitro* results.

Line 86

Herein we describe the first chemical synthesis of Chlorosphaerolactylate B. Chlorosphaerolactylate B was the natural extracted halogenated lactylates with best antimicrobial (both antibiofilm and antibacterial) activity.

Line 111

Antibacterial activity: The *in vitro* MIC value of synthetic Chlorosphaerolactylate B against the *S. aureus* strain S54F9 was 256 mg/L. Positive and negative controls performed as expected. Antibiofilm activity: The *in vitro* MBIC value of synthetic Chlorosphaerolactylate B against the Coagulase Negative *Staphylococcus* strain FI31 was 74 mg/L. No antibiofilm activity was registered for intermediate products (**3** and **4**). Positive and negative controls performed as expected.

The *in vitro* results are just a single value for MIC and MBIC, respectively, and we find it difficult to express that in a figure. However, we have changed “all controls performed as expected” to “Positive and negative controls performed as expected, i.e. growth and no growth, respectively.

Additionally, even though the authors are working to *S. aureus*, it is difficult to suggest that there is antibiofilm activity in the in vivo since the presence/quantity of the biofilm in the porcine model was not actually determined, only microbial presence per se. 
Thank you for this observation. In the porcine model we use sonication of implants as an important measurement of biofilm formation. According to many acknowledge authors in the field bacterial growth on implants represents biofilm formation. Previously, we have shown that biofilm is formed on the implants and in the surrounding tissue using electron microscopy and histochemistry. In these studies, biofilm was defined as bacterial aggregates surrounded by substantial extracellular matrix. The following lines has been included in the manuscript:

Line 118

The experiment applied an *in vivo* susceptibility test for prevention of biofilm formation in tissue and on implants (23). The test utilizes the porcine osteomyelitis model described by Jensen *et al.* 2017 (19). In this model, biofilm is understood as bacterial aggregates surrounded by extracellular matrix and the presence of biofilm has been demonstrated using electron microscopy, immunohistochemistry and in-situ hybridization (9, 24).

Additionally, the discussion could be briefly expanded to include a comparison with other products against the used *S. aureus* to better understand its real potential.

The following lines has been included.

Line 188

Other products against the used *S. aureus* strain entails only the aminoglycoside gentamicin which commonly is used in revision surgery of bone infections in humans (27). In a study based on the porcine IAO model gentamicin was used as active ingredient in a local bone void filler following revision surgery (28). However, a week after revision the infection was reestablished properly due to remnant biofilms. This indicate the need for development of more biofilm efficient antibiotics.

Line 66: “with rising antimicrobial resistance”. It should be “of rising antimicrobial resistance”. 
This has now been corrected

Line 73-74: Some references could be provided here. 
A new reference has been added

Line 73 ref no 12

Lau, J. S. Y., Bhatt, S., Streitberg, R., Bryant, M., Korman, T. M., Woolley, I. (2019). Surveillance of life-long antibiotics—A cross-sectional cohort study assessing patient attitudes and understanding of long-term antibiotic consumption. *Infect. Dis. Health* 24, 179–186. doi: 10.1016/j.idh.2019.05.002

Line 78: References from cyanobacteria are not included. See for instance:
Thank you for providing these references. They have now been included.

Line 77 ref no 14 +15

Antunes, J., Pereira, S., Ribeiro, T., Plowman, J. E., Thomas, A., Clerens, S., Campos, A., Vasconcelos, V., Almeida, J. R. (2019). A Multi-Bioassay Integrated Approach to Assess the Antifouling Potential of the Cyanobacterial Metabolites Portoamides. *Mar. Drugs* 17, 111. doi: 10.3390/md17020111

Santhakumari, S., Kannappan, A., Pandian, S. K. Thajuddin N, Rajendran, R. B., Ravi, V. A. (2016). Inhibitory effect of marine cyanobacterial extract on biofilm formation and virulence factor production of bacterial pathogens causing vibriosis in aquaculture. *J. Appl. Phycol*. 28, 313–324. doi: 10.1007/s10811-015-0554-0

Line 87-88: This sentence is somewhat confusing, please rephrase.

This line been rewritten to:

Line 86. Herein we describe the first chemical synthesis of Chlorosphaerolactylate B. Chlorosphaerolactylate B was the natural extracted halogenated lactylates with best antimicrobial (both antibiofilm and antibacterial) activity. Furthermore, we confirmed the antimicrobial activity of the synthetic compound both *in vitro* and *in vivo* by using a porcine model of implant associated osteomyelitis (IAO) infected with *S. aureus* strain S54F9 (18, 19).

Line 112: “All controls performed as expected” Please elaborate further.

The following has been included:

Antibacterial activity: The *in vitro* MIC value of synthetic Chlorosphaerolactylate B against the *S. aureus* strain S54F9 was 256 mg/L. Positive and negative controls performed as expected, i.e. growth and no growth, respectively. Antibiofilm activity: The *in vitro* MBIC value of synthetic Chlorosphaerolactylate B against the Coagulase Negative *Staphylococcus* strain FI31 was 74 mg/L. No antibiofilm activity was registered for intermediate products (**3** and **4**). Positive and negative controls performed as expected.

Line 122: “All inoculations were successful” How was this determined? 
Thank you for pointing out. We mean that all inoculations occurred according to the protocol. The line has been changed to: Line 128. All inoculations were successful, i.e. the protocol was fulfilled

Line 134: What is meant by “generally”? 
Nothing is meant with generally and therefore; it has now been removed

Line 141: Which sort of stable environment and why is this the likeliest hypothesis?
Thank you for asking. The contaminating bacteria are natural occurring on the skin of pigs and within their oral cavity and feces. As the pigs are housed in groups where they are licking each other and the equipment and also, they are housed with a lot of straw, this is the likeliest explanation.

hLine 148: “having large PIBA values”. Were any statistical tests conducted to determine the significance of these differences? Or for any other data in the manuscript?

Good comment. We choose not to perform statistics due to the low number of animals and instead show all the individual values in the figures. It has been emphasized in the conclusion that this study is hypothesis generating for further investigations. But it provides the first indication for the use of this new group of antibiotics.

Line 230

The present study is descriptive and hypothesis generating for further investigations of this new discovered type of antimicrobials. More *in vivo* studies of synthetic Chlorosphaerolactylate B are required in order to disclose dose-response profile, long-time exposure effects or understanding the mechanisms of actions

Line 152: Why is “groups” in capital letters here? 
This has now been corrected

Line 162: “controls performed as expected” How so? Control results could also be shown.

Thank you for the comment. Normally controls are not shown for immunohistochemistry. By “as expected” we mean that the positive controls show IHC staining of a component known to express the antigen and negative (nonsense) controls show no IHC staining at all. The line has been changed to: “All positive and nonsense IHC controls performed as expected, i.e. with positive staining and no staining, respectively”. (line 169)

Line 177: “and MBIC values of the synthetic compound were found lower than the values observed for the natural”. How does this compare to standard commercial products? This could be added in the manuscript.

This difference may be attributed to the mixture of potential isoforms in the natural version of Chlorosphaerolactylate B. This is mentioned in the discussion (line 184). We think this the relevant information needed on this topic.

Line 268: “S. aureus S54F9, spa type t1333” Why was this strain selected? 
The strain was selected because we know how it behaves in the porcine model. Furthermore, the strain has been comprehensively investigated and we know it produce biofilm. A reference on this has now been included.

The line (319) is now: The strain is highly virulent and has genes encoding several toxins, including phage-associated enterotoxin, exotoxins and superantigen (38). Furthermore, the strain produces biofilm (24).

New reference 24 is: Jensen, L. K., Henriksen, N. L., Bjarnsholt, T., Kragh, K. N., Jensen, H. E. 2018. Combined Staining Techniques for Demonstration of Staphylococcus aureus Biofilm in Routine Histopathology, *J. Bone Joint Infect.* 3, 27–36. doi: 10.7150/jbji.22799

Line 275: “The antibiofilm activity…” There are no figures nor tables presenting these results, they should be provided. 
See general comment in the beginning of this document

Line 278: “96-well microtiter plates”. Please add a reference for this product.

This has now been included (line 285 + 291). Thermo Fisher scientific, Barcelona, Spain

Line 290: “Jensen et al.” Year of paper is missing. 
This has now been corrected

Line 291: Why only females were selected. An explanation and/or reference should be provided. 
Female pigs were used in order to reduce variability between the animals and due to group housing. The reviewer is right, in further experiments with larger group sizes both sexes should be included.

Line 307: Only female pigs were used in order to reduce variance within and between groups.

Line 308: Why was this value (283 mg) for the inoculum selected?

A value of 283 mg is close to the dose of gentamicin (in terms of times the MIC or MBIC value) used in a previous study where the premixing inoculation method of the model was established. This is pointed out in the discussion Line 201: The synthetic Chlorosphaerolactylate B concentration used *in vivo* was much higher than the estimated MBIC value. The used concentration of 159.346,8 times the MBIC value was chosen based on the original data from the applied *in vivo* biofilm susceptibility study (23). In that study, 160.000 times the MIC value for gentamicin was needed to prevent bacterial growth on both the implant surface and within the surrounding tissue of the porcine IAO model (23).

Line 310: Comma is missing after “afterwards”. 
This has now been corrected

Line 316: “were inspected for signs of inflammation and infection”. How was this conducted? 
Good comment. This was conducted with histology by looking for inflammatory cells, bacteria or cell damages like necrosis. The line has been changed to:

Line 340: (see histology).

Line 366: Lung, liver, kidney, and spleen slides were evaluated for infection and inflammation in terms of signs of necrosis and cellular degeneration, systemic spreading of the inoculated bacteria and infiltration of inflammatory cells.

Reviewer 3

One of the problems to be dealt with regarding antibiotic resistance, which is one of the biggest problems of our time, is biofilms. In this study, Chlorosphaerolactylate B compound, which was determined as an output of the NOMORFILM project, was obtained synthetically and its in vivo properties were investigated. A very good study sheds light on the potential use of the halometabolite Chlorosphaerolactylate B. However, I suggest that some empirical parts be explained and discussed.

1. Why were experiments started 5 days after inoculation of pigs with S. aureus (and other groups)?

This is a very relevant question – thank you.

The infection time of 5 days was chosen, as this is the infection-time used in the applied *in vivo* susceptibility test (23). It has been shown several times that a fulminant bone infection with biofilm formation is formed after 5 days within the model, i.e., pathological manifestations as seen in humans with bone infections are present. We aim for a short infection time as possible due to animal welfare.

Line 122: It has been shown several times that a fulminant bone infection with biofilm formation is formed after 5 days within the model, i.e. pathological manifestations as seen in humans with bone infections are present (18,19).

2. In the manuscript, 1% Crystal Violet was used in antibiofilm experiments. However, this dye concentration is too high.

Thank you for the comment.

This concentration has been used for many years in the lab where the antibiofilm study was performed. As the same concentration was used in the positive and negative controls, the differences found are comparative.

3. According to the diagram given in Figure 2, it may be possible that Chlorosphaerolactylate B and S. aureus were previously combined, and S. aureus may have died before implantation. How long was the wait during the process here? should be explained.

Thank you for this relevant observation

The mixing time was 60 seconds. This is the mixing time described in the applied *in vivo* susceptibility test (23). The same suspension as used for inoculation (10 microL with *S. aureus* and Chlorosphaerolactylate B mixed for 60 seconds) was applied to a petri dish with 5% blood ager. This demonstrated that *S. aureus* was still viable after mixing.

The following lines has been added:

Line 328: Furthermore, 10 μL mixing solutions (*S. aureus* and Chlorosphaerolactylate B mixed for 60 seconds) was also applied to a petri dish with blood ager. This demonstrated that *S. aureus* was still viable after mixing.

Kind regards

Louise Kruse Jensen and Fernando López Ortiz
